# Impact of add-on laboratory testing at an academic medical center: a five year retrospective study

**DOI:** 10.1186/s12907-015-0011-7

**Published:** 2015-06-07

**Authors:** Louis S. Nelson, Scott R. Davis, Robert M. Humble, Jeff Kulhavy, Dean R. Aman, Matthew D. Krasowski

**Affiliations:** Department of Pathology, University of Iowa Hospitals and Clinics, Iowa City, IA 52242 USA; Hospital Computing Information Services, University of Iowa Hospitals and Clinics, Iowa City, IA 52242 USA

**Keywords:** Clinical chemistry tests, Clinical laboratory information services, Clinical laboratory services, Hematology, Laboratory automation, Robotics

## Abstract

**Background:**

Clinical laboratories frequently receive orders to perform additional tests on existing specimens (‘add-ons’). Previous studies have examined add-on ordering patterns over short periods of time. The objective of this study was to analyze add-on ordering patterns over an extended time period. We also analyzed the impact of a robotic specimen archival/retrieval system on add-on testing procedure and manual effort.

**Methods:**

In this retrospective study at an academic medical center, electronic health records from were searched to obtain all add-on orders that were placed in the time period of May 2, 2009 to December 31, 2014.

**Results:**

During the time period of retrospective study, 880,359 add-on tests were ordered on 96,244 different patients. Add-on testing comprised 3.3 % of total test volumes. There were 443,411 unique ordering instances, leading to an average of 1.99 add-on tests per instance. Some patients had multiple episodes of add-on test orders at different points in time, leading to an average of 9.15 add-on tests per patient. The majority of add-on orders were for chemistry tests (78.8 % of total add-ons) with the next most frequent being hematology and coagulation tests (11.2 % of total add-ons). Inpatient orders accounted for 66.8 % of total add-on orders, while the emergency department and outpatient clinics had 14.8 % and 18.4 % of total add-on orders, respectively. The majority of add-ons were placed within 8 hours (87.3 %) and nearly all by 24 hours (96.8 %). Nearly 100 % of add-on orders within the emergency department were placed within 8 hours. The introduction of a robotic specimen archival/retrieval unit saved an average of 2.75 minutes of laboratory staff manual time per unique add-on order. This translates to 24.1 hours/day less manual effort in dealing with add-on orders.

**Conclusion:**

Our study reflects the previous literature in showing that add-on orders significantly impact the workload of the clinical laboratory. The majority of add-on orders are clinical chemistry tests, and most add-on orders occur within 24 hours of original specimen collection. Robotic specimen archival/retrieval units can reduce manual effort in the clinical laboratory associated with add-on orders.

## Background

Clinical laboratories frequently receive orders to perform additional tests on existing specimens (‘add-ons’). Melanson et al. in 2004 was the first published report analyzing the operational impact of add-on testing, demonstrating patterns of misutilization (e.g., failure to follow laboratory testing algorithms in institutional chest pain protocols) in a significant fraction of add-on orders [1]. A follow-up study in 2006 compared add-on testing between two academic hospitals, showing similarities in add-on ordering patterns and proposing strategies to improve the process [2]. There have been several other studies on add-on test ordering, each analyzing less than one month of add-on orders [3–5].

In this study at an academic medical center, we retrospectively analyzed add-on testing data over a five and a half year period (May 2009- Dec 2014). This allowed for the analysis of add-on ordering trends over a much longer period of time than in previous studies. Also, during this time period, the core clinical laboratory of the institution introduced a robotic archival specimen retrieval system that changed the add-on testing procedure. We analyzed the impact of this unit on add-on testing procedure and manual workload.

## Methods

### Institutional setting

The study was approved by the University of Iowa Institutional Review Board as a retrospective study covering the time period from May 2, 2009- December 31, 2014. In this large retrospective study, there was waiver of informed consent and authorization approved by the Institutional Review Board for all subjects. The institution in this study is the University of Iowa Hospitals and Clinics (UIHC), a 730 bed academic medical center that includes an emergency department (ED) with level one trauma capability, adult and pediatric inpatient floors, and multiple intensive care units (ICUs; neonatal, pediatric, medical, cardiovascular, and neurologic/surgical). Outpatient services are located at the main medical campus in Iowa City, IA, as well as at a multispecialty outpatient facility located three miles away. Smaller primary care clinics are located throughout the local region. A core clinical laboratory within the Department of Pathology provides clinical chemistry and hematopathology testing. Two critical care laboratories (one located near the main operating rooms and another embedded within the neonatal ICU) perform blood gas and activated clotting time testing. There are also separate clinical laboratories for anatomic pathology, blood center, and microbiology/molecular pathology located within the main medical campus.

### Hospital and laboratory informatics

The electronic health record (EHR) for UIHC was Epic (Epic Systems, Inc, Madison, WI). Computerized provider order entry (CPOE) is available in Epic to licensed independent providers. Add-on orders can be placed within the EHR by CPOE or by calling the laboratory. Throughout the period of retrospective study, providers were directed, when feasible, to place orders within the EHR and limit the number of verbal orders requiring laboratory-initiated testing orders. In general, chemistry and hematology tests are all orderable individually. However, there are some panels built in Epic: basic metabolic panel with total calcium (BMP; sodium, chloride, carbon dioxide, potassium, blood urea nitrogen, creatinine, glucose, total calcium), electrolyte panel (sodium, chloride, carbon dioxide, potassium), complete blood count (CBC; white blood cell count, hemoglobin, hematocrit, red blood cell count, platelet count), and lipid panel (total cholesterol, high-density lipoprotein, triglycerides, calculated low-density lipoprotein). For the purposes of analysis in this manuscript, panels were broken apart into individual tests except where described otherwise. Categories of testing were also defined (Table 1) to provide better comparison to other published studies on add-on testing [1, 2, 5].

**Table 1:** Abbreviations for assay categories

Abbreviation	Full Name	Test(s) Included
A1C	Hemoglobin A1C	Hemoglobin A1C
ANEMIA	Anemia Testing	Iron, total iron-binding capacity, ferritin, folate, vitamin B_12_
BILD	Bilirubin, Direct	Direct (conjugated) bilirubin
BMP	Basic Metabolic Panel	Sodium, potassium, chloride, carbon dioxide, blood urea nitrogen, creatinine, glucose, and calcium
CARDIAC	Cardiac Markers	Creatine kinase-MB, troponin T, *N*-terminal B-type natriuretic peptide
CBC	Complete Blood Count	White blood cell count, red blood cell count, hemoglobin, hematocrit, platelet count
CRP	C-Reactive Protein	C-Reactive Protein
DIFF	Differential	White blood cell differential
ENDO	Endocrinology Testing	Thyroid-stimulating hormone, thyroxine (T_4_) – total and free, triiodothyronine (T_3_) – total and free, cortisol, testosterone, and 25-hydroxyvitamin D
ESR	Erythrocyte Sedimentation Rate	Erythrocyte sedimentation rate
GASES	Blood Gas Analyzer Laboratory Studies^a^	Lactic acid, potassium, glucose, hemoglobin, hematocrit, sodium, chloride, ionized calcium, pO_2_, pCO_2_, oxygen saturation, methemoglobin, carboxyhemoglobin
HAPT	Haptoglobin	Haptoglobin
HEPC	Hepatitis C Antibody	Hepatitis C antibody
HBSG	Hepatitis B Surface Antigen	Hepatitis B surface antigen
LFP	Liver Function Panel	Albumin, alkaline phosphatase, total bilirubin, total protein, alanine aminotransferase, aspartate aminotransferase, and γ-glutamyltranspeptidase
LDH	Lactate Dehydrogenase	Lactate dehydrogenase
REFERENCE	Reference Laboratory Testing	All testing referred to external reference laboratory
OSMO	Osmolality	Serum and urine osmolality
PO4MG	Phosphorus and Magnesium	Phosphorus, magnesium
PREALB	Prealbumin	Prealbumin
PT/INR	Prothrombin Time/INR	Prothrombin time/International normalized ratio
PTT	Partial Thromboplastin Time	Partial thromboplastin time
RETIC	Reticulocytes	Reticulocytes
TAP	Toxic Alcohol Panel	Sodium, glucose, blood urea nitrogen, osmolality, ethanol
URIC	Uric Acid	Uric acid

^a^These are studies using whole blood and not plasma or serum

The laboratory information system (LIS) for all UIHC pathology laboratories until August 2, 2014 was Cerner (Kansas City, MO, USA) “Classic”, currently version 015. On August 2, 2014, the clinical pathology laboratories switched to Epic Beaker as the LIS, retaining Cerner as the LIS for anatomic pathology, blood center, and some parts of hematopathology and molecular pathology. The switch to Epic Beaker allowed for accurate capture of the timing of add-on orders relative to when the original specimen was received in the laboratory. During this nearly 5 month period (August 2 to December 31, 2014), there were 56,389 add-on orders with complete time data.

### Laboratory instrumentation and add-on testing procedures

**Fig. 1:**
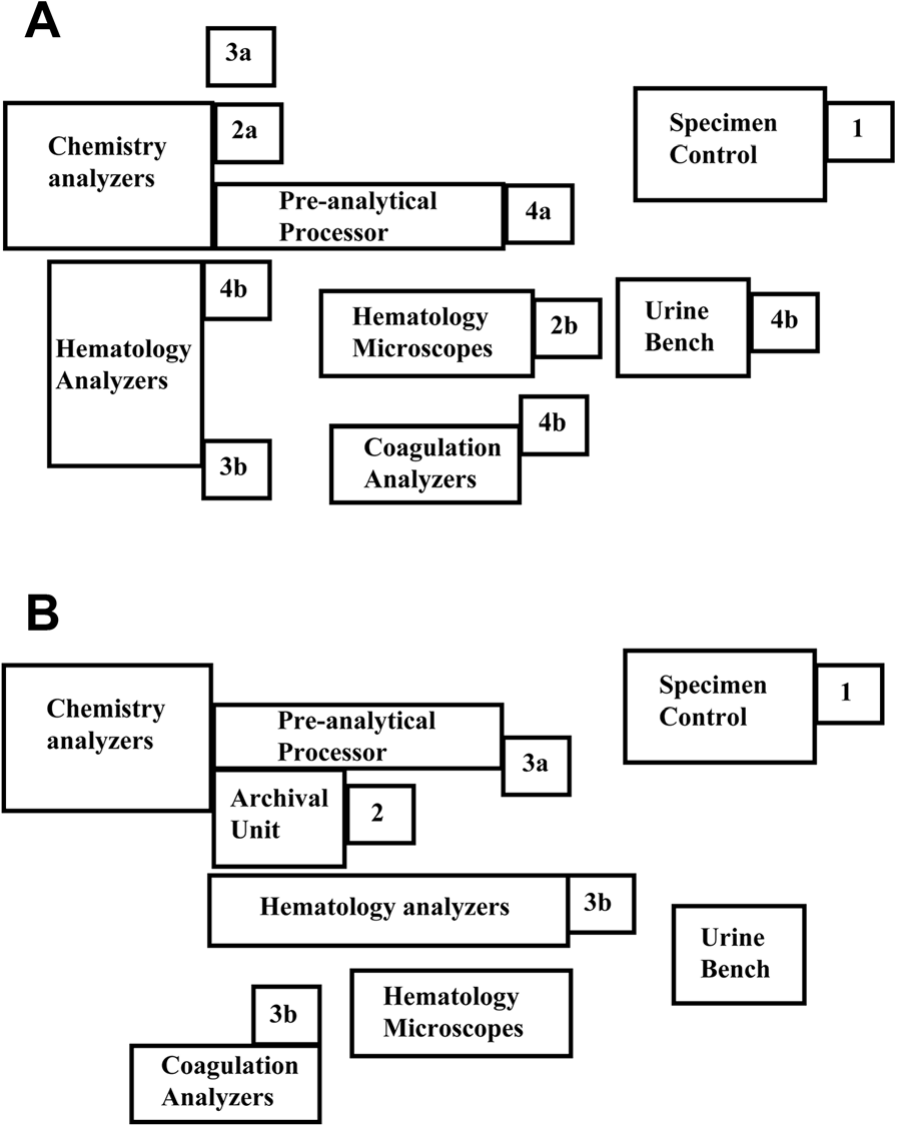
Add-on Testing Procedure. **a** Layout of the core laboratory prior to the automated specimen archival/retrieval unit. Add-on orders generate a print-out in the core laboratory with the testing information and patient demographics (1). A laboratory assistant reviews the print-out. If an add-on can be performed, the assistant prints an additional label with test information to a designated area of the laboratory (e.g., chemistry, 2a, or hematology, 2b) depending on the add-on order. A technologist enters in accession number into the computer program that tracks the archival rack and position for the specimen. Then technologist retrieves the specimen from the archival rack (3a or 3b) or from the refrigerator. The technologist loads specimen on proper analyzer (4a, 4b). **b** Layout of the core laboratory after the automated specimen archival/retrieval unit. Similar to above, add-on orders generate a print-out in the core laboratory that is reviewed by laboratory assistant (1). If an add-on can be performed, the assistant then uses a computer program to request the specimen be retrieved from the archiver (1). The archiver then locates the specimen and dispenses it (2). The assistant retrieves the specimen from archiver and loads specimen on to proper analyzer (3a or 3b)

The instrumentation and informatics within the core laboratory of UIHC has been described in detail in previous reports [6, 7]. Throughout the time period of retrospective analysis, the main chemistry instrumentation in the core laboratory was from Roche Diagnostics (Indianapolis, IN, USA). Front-end automation was provided by a Modular Pre-Analytic (MPA)-7 unit. In February 2014, the core laboratory went live with a Roche Diagnostics P701 automated archival/retrieval system. This system changed the add-on process (Fig. 1). Originally, specimens were placed into archival racks by the instrument flexible sample sorters. The most recent racks were kept near the instruments. The racks were then archived manually to a set of refrigerators for storage for 3 to 5 days (dependent on available refrigerator space and number of specimens). The P701 automated the sample storage and retrieval process (Fig. 1b), eliminating manual steps.

The manual effort involved from the moment the add-on is printed into the laboratory to the time the sample is loaded on the proper analyzer averaged approximately 3.5 minutes prior to introduction of the P701 (composed in large part in retrieving specimens from racks near instruments or in the refrigerators), and 45 seconds once the P701 was implemented. In the prior system of manual archiving and retrieval, instances where samples were archived improperly could significantly delay the process.

## Results

### Timing trends in add-on testing

**Fig. 2:**
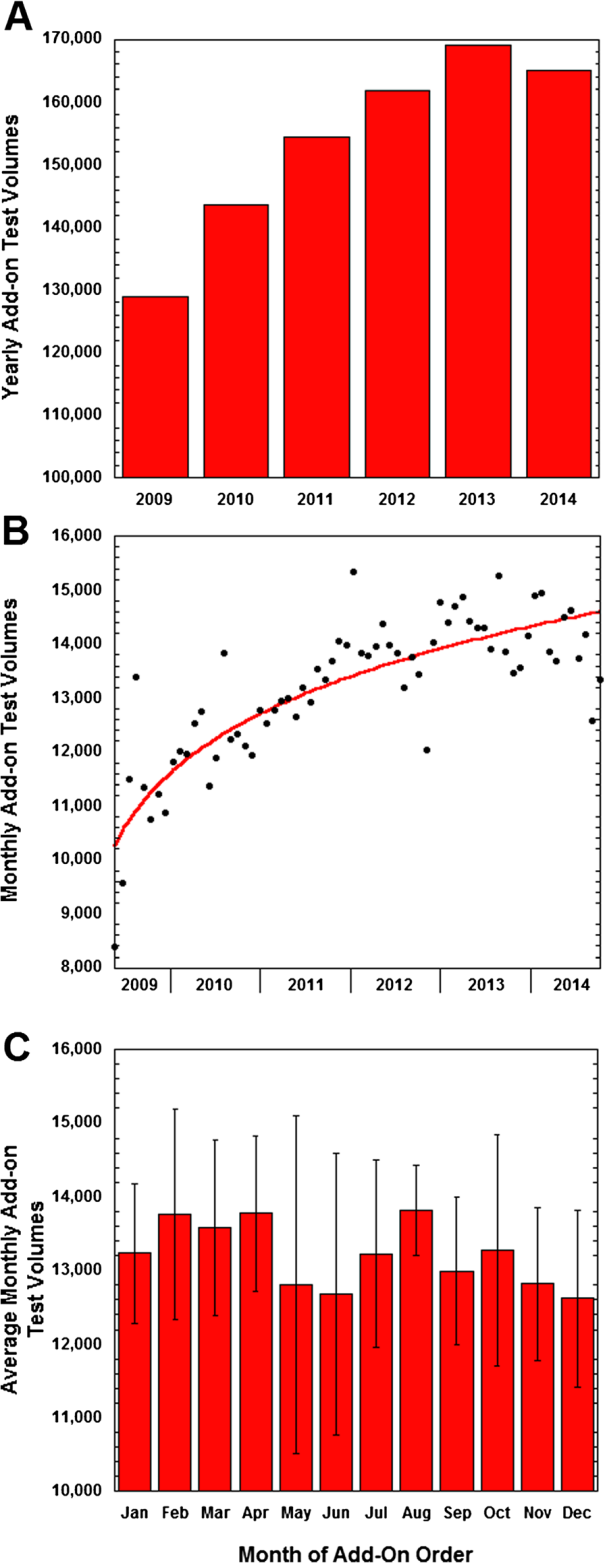
Add-on Order Volumes. **a** Yearly add-on testing volumes from 2009 – 2014. The data in 2009 is normalized to an entire year based on order volume from May 2, 2009 to end of that calendar year. **b** Monthly add-on testing volumes spanning 2009 – 2014. **c** Add-on test volumes per month

In the time period of retrospective study (May 2, 2009 to December 31, 2014), there were a total of 880,359 add-on orders at UIHC. This comprised 3.3 % of the total laboratory test volume performed within the clinical laboratories. The total number of add-on orders increased every year from 2009 to 2013 and then decreased slightly in 2014 (Fig. 2a, b). Fig. 2c shows the variation of add-on orders by month. The inpatient population had the majority of add-on orders (66.8 %), while the ED and outpatient clinics accounted for 14.8 % and 18.4 % of add-on orders, respectively.

**Fig. 3:**
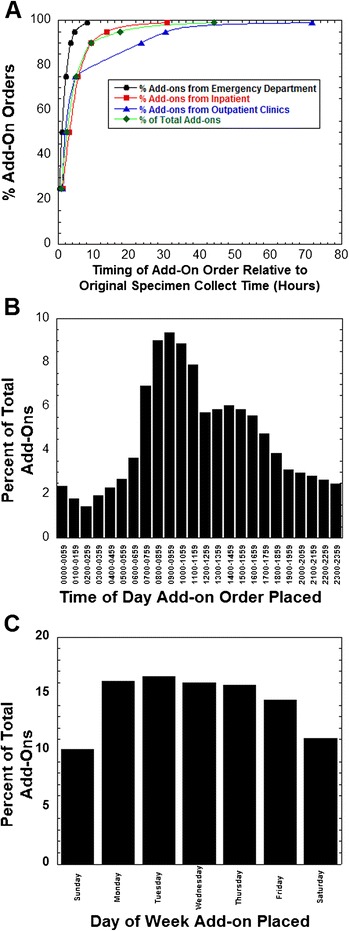
Timing of Add-on Orders. **a** Timing of add-on orders relative to original specimen collect time. The data is broken down into orders originating from emergency department (ED), inpatient units (including intensive care units), outpatient clinics, and all data. **b** Time of day add-on order was placed broken into one hour intervals. **c** Day of week add-on order was placed

Capture of the exact timing of add-on orders relative to initial specimen collect was only possible with the new LIS (August 2, 2014 – December 31, 2014; complete data available on 56,389 add-on orders). Fig. 3a plots the timing of add-on orders, showing the percentage within periods of time. The majority of add-ons were placed within 8 hours (87.3 %) and nearly all by 24 hours (96.8 %). The timing of ordering varied by patient location. Add-on orders placed for patients in the ED were generally closer to original specimen collect time as compared to inpatient units and outpatient clinics. Nearly 100 % of add-on orders within the ED were placed within 8 hours. Timing of add-on orders for inpatient units and outpatient clinics were similar, except that there was a longer tail of add-on orders placed 24 hours or more later than original specimen collect in the outpatient population (Fig. 3a).

The peak times for add-on order placement were between 08:00 – 12:00, with 47.8 % of add-ons ordered between 07:00 and 13:00 (Fig. 3b). Add-on orders were more frequent during weekdays, with Saturdays and Sundays only accounting for slightly more than 20 % of total add-on volume (Fig. 3c).

### Distribution of add-on testing by category of testing

**Fig. 4:**
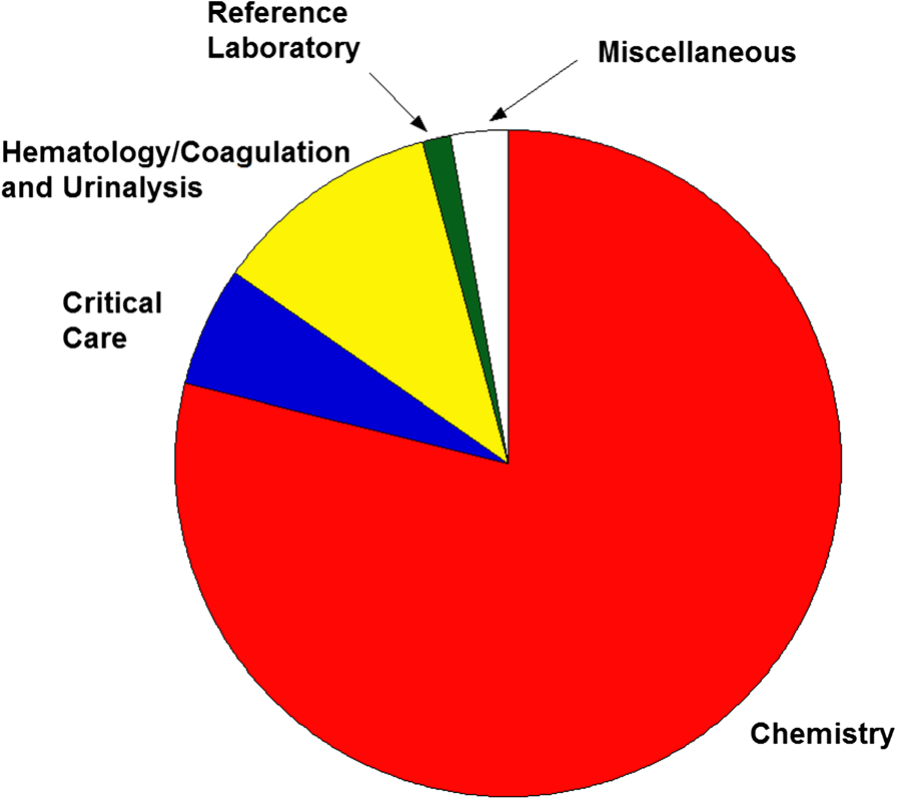
Breakdown of Add-on Order Volumes by Category of Testing. The categories of testing are: Clinical Chemistry (performed in core laboratory), Hematology/Coagulation and Urinalysis (performed in core laboratory), Critical Care (blood gases, whole blood electrolytes, and other testing performed in satellite critical care laboratories), Reference Laboratory (testing referred to outside laboratories), and Miscellaneous (all testing not fitting into other categories)

Figure 4 shows a breakdown of add-on testing by category of testing. The majority of the add-on tests were chemistry tests (78.8 % of total add-on orders comprising 3.3 % of overall chemistry test volumes), with the most frequent being within the following categories (all percentages are of the total overall add-on orders): LFP (25.6 %), BMP (11.7 %), MGPO4 (7.0 %), Cardiac (4.6 %), and Anemia (3.2 %). Hematology and coagulation tests were the next most frequent areas of add-on testing. The most frequent hematology and coagulation add-ons were in the following categories (all percentages are of the total overall add-on orders): CBC (2.70 %), Diff (2.19 %), PTT (1.19 %), PT/INR (1.71 %), and ESR (0.85 %). Within the critical care laboratories, the most frequent add-on orders were the following tests performed on blood gas analyzers using whole blood specimens: lactic acid (0.97 %), potassium (0.78 %), glucose (0.62 %), hemoglobin/hematocrit (0.49 %), and sodium (0.49 %). Urinalysis-related tests accounted for less than 1 % of total add-ons. For the critical care laboratory tests, in most cases the parameters had already been determined as part of a cartridge of testing on the blood gas analyzers; results were suppressed from reporting to the LIS if not ordered by the provider. Thus, the add-on order for these tests required only that staff perform the computer steps necessary to release the previously unordered test from the instrument to the LIS.

Table 2 summarizes the most frequent add-on orders broken down into categories of testing. LFP, BMP, MGPO4, ENDO, and CARDIAC ranked in the top ten most frequent add-on orders overall and also individually for inpatient units, ED, and outpatient clinics. Not surprisingly, GASES were an infrequent add-on order in the outpatient clinics but were the fourth most common overall add-on order. A1C was more frequently added-on in the outpatient clinics than from the ED or inpatient units. Table 3 lists the most frequent individual tests added-on. The top five most frequent individual add-on tests were magnesium (4.0 %), albumin (3.8 %), alanine aminotransferase (3.8 %), aspartate aminotransferase (3.8 %), and total bilirubin (3.7 %). Table 3 also lists the percentage of times each individual test was ordered as an add-on. For six of the tests (amylase, creatine kinase, hemoglobin A1C, lactate dehydrogenase, lipase, troponin T), over 10 % of orders for that particular test were placed as add-ons.

**Table 2:** Most frequently ordered add-on test categories by ordering location

	Inpatient Unit		Emergency Department		Outpatient Clinics		All Locations	
Test Category	%	Rank	%	Rank	%	Rank	%	Rank
LFP	28.79 %	1	29.86 %	1	16.91 %	1	26.76 %	1
BMP	12.00 %	2	10.14 %	2	15.12 %	2	12.30 %	2
MGPO4	7.41 %	4	4.02 %	4	2.87 %	5	6.08 %	3
GASES	8.41 %	3	2.51 %	9	0.10 %	19	6.01 %	4
ENDO	3.30 %	6	3.09 %	6	8.81 %	3	4.28 %	5
CARDIAC	4.19 %	5	7.25 %	3	2.09%	7	4.25 %	6
ANEMIA	2.66 %	7	0.78 %	18	6.67 %	4	3.12 %	7
DIFF	2.40 %	8	1.73 %	15	1.78 %	9	2.19 %	8
CBC	2.24 %	9	1.83 %	14	2.00 %	8	2.14 %	9
PT/INR	1.43 %	12	3.57 %	5	0.88 %	13	1.65 %	10
LIPASE	1.49 %	11	3.01 %	8	0.77 %	15	1.58 %	11
A1C	1.33 %	14	0.95 %	17	2.52 %	6	1.49 %	12
CRP	1.29 %	15	2.22 %	10	1.39 %	11	1.45 %	13
LDH	1.50 %	10	0.64 %	19	1.43 %	10	1.36 %	14
AMYLASE	1.37 %	13	2.02 %	12	0.68 %	16	1.34 %	15
PTT	0.97 %	16	3.03 %	7	0.19 %	17	1.13 %	16
ESR	0.61 %	18	2.00 %	13	0.78 %	13	0.85 %	17
BILD	0.94 %	17	0.59 %	20	0.92 %	12	0.85 %	18
D-DIMER	0.24 %	19	2.07 %	11	0.11 %	18	0.48 %	19
TAP	0.16 %	20	1.61 %	16	0.03 %	20	0.35 %	20
Total	82.7 %		82.9 %		66.1 %		79.7 %

**Table 3:** Most frequently ordered individual add-on tests

Test	% of Total Add-on Orders	% of Times Test Ordered as Add-on (vs. Routine)
Magnesium	4.0	5.9
Albumin	3.8	6.5
Alanine aminotransferase	3.8	5.4
Aspartate aminotransferase	3.8	5.5
Bilirubin, total	3.7	6.4
Alkaline phosphatase	3.4	5.8
Gamma-glutamyltransferase	3.2	4.7
Total protein	3.1	6.3
Phosphorus	3.0	6.9
Troponin T	2.2	14.5
Complete blood count	2.1	1.5
Automated white blood cell differential	2.1	2.3
Creatinine	1.7	1.1
Potassium	1.6	1.1
Thyroid stimulating hormone with reflex to free thyroxine	1.6	9.6
Lipase	1.6	15.9
Hemoglobin A1C	1.5	10.2
Prothrombin time/International normalized ratio	1.5	1.8
Basic metabolic panel with calcium	1.5	1.1
C-Reactive protein	1.4	8.1
Lactic acid dehydrogenase	1.4	10.7
Creatine kinase, total	1.3	22.7
Amylase	1.3	15.3
Blood urea nitrogen	1.3	0.9
Calcium, total	1.2	1.3

Reference laboratory testing comprised only 1.4 % of total add-ons, with no single test accounting for more than 0.05 % of total add-ons (Table 4). There were 618 different reference laboratory tests available in the EHR test menu that were ordered at least once as an add-on in the time period of retrospective study. This included 193 orders of “Miscellaneous Test”, an order option in the EHR for reference laboratory testing not built in the EHR test menu. Our analysis does not capture attempts to order reference laboratory testing that could not be completed due to lack of suitable existing specimen.

**Table 4:** Most frequently ordered reference laboratory add-on tests

Test	% of Total Add-on Orders
*Helicobacter pylori* antibody, IgG	0.050 %
Mitochondrial M2 antibodies	0.049 %
Hepatitis C virus quantitative PCR	0.033 %
IgG subclasses	0.022 %
Miscellaneous test^a^	0.019 %
Vitamin D, 1,25-dihydroxy	0.018 %
Aldosterone, serum	0.017 %
Alpha-1-antitrypsin, phenotyping	0.013 %
Lyme disease antibodies, IgG and IgM	0.013 %
*Histoplasma* antigen, urine	0.013 %

^a^Covers any test ordered that is not on list of laboratory tests built in electronic medical record

### Workload impact of add-on testing

Table 5 summarizes the workload of add-on testing over the period of retrospective study. Of the 880,359 add-on orders, there were 443,411 unique ordering instances, leading to an average of 1.99 add-on tests per instance. Some patients had multiple episodes of add-on test orders at different points in time (e.g., during different days of a multi-day inpatient encounter), leading to an average of 9.15 add-on tests per patient. The introduction of the robotic specimen archival/retrieval unit saved an average of 2.75 mins of laboratory staff manual time per unique ordering instance (Table 5). This translates to 24.1 hr/day less manual effort in dealing with add-on orders.

**Table 5:** Summary of add-on tests

Total number of add-ons	880,359
Unique ordering instances^1^	443,411
Number of patients	96,244
Average number of add-on tests per unique ordering instance	1.99
Average number of add-on tests per patient^2^	9.15
Estimated hours per day of manual effort for add-on testing prior to introduction of robotic archival storage/retrieval unit	30.7
Estimated hours per day of manual effort for add-on testing with use of robotic archival storage/retrieval unit	6.6

^1^These are unique add-on orders at a specific time

^2^This includes all add-on tests per each patient, which may span multiple unique ordering instances

## Discussion

Add-on testing can occupy a significant amount of clinical laboratory resources [1, 3–5]. The main challenges are storage of specimens and the labor involved in retrieving specimens for further testing [1, 5]. Add-on testing can theoretically serve a useful purpose in allowing for thoughtful ordering of additional testing based on initial laboratory test results or other clinical data. On the other hand, add-on testing can also be a result of disorganized ordering practices (e.g., neglecting to order essential laboratory testing upfront) or even misutilization of testing, as has been shown in previous studies [1, 2]. Our analysis does not capture attempts at add-on testing that could not be completed due to lack of existing specimens or add-on testing that was duplicate to previously ordered testing (e.g., hemoglobin/hematocrit placed as add-on when CBC already performed). We have analyzed duplicate testing as an example of misutilization in a previous study [6].

The results of this study are similar to previous studies in showing that the majority of add-ons are clinical chemistry tests [2, 4, 5]. As with previous studies, LFP, BMP, MGPO4, and CARDIAC were in the top tier of ordered add-on tests. Hematology and coagulation testing only accounted for 11.2 % of total add-ons; however, add-ons comprised 5.6 % of the overall hematology/coagulation test volumes. Four common tests (CBC, DIFF, PT/INR, and PT) accounted for over two-thirds of the hematology and coagulation add-on tests.

In the present study, the 5.86 % percent of add-ons for the critical care laboratories posed the least manual effort because in most cases the parameters had already been determined but were suppressed from reporting to the LIS if not ordered by the provider. Therefore, most critical care laboratory add-ons only needed to be sent from instrument to LIS rather than having to locate the sample and analyze it again (assuming specimen would even be viable at that point). Reference laboratory add-ons were only a small fraction of total add-ons but spanned a wide variety of tests. No single reference laboratory test exceeded 0.05 % of total add-ons. These results are comparable to previous studies [2, 5]. Add-on requests for reference laboratory tests not built in the LIS or EHR may entail extra work in first determining the specimen requirements for the requested test and then checking if pre-existing specimens can be used.

Similar to previous studies, our study shows a high fraction of add-ons ordered within eight hours of original specimen collect time [1, 2, 5]. The ED tended to order add-ons more quickly compared to inpatient units and outpatient clinics. Only a small fraction of add-on orders were placed more than 24 hours after original specimen collection. Less than 1 % of total add-ons occurred more than 48 hours after specimen collection. Outpatient clinics accounted for the majority of add-on orders submitted more than 24 hours after original specimen collection, a finding similar to a previous study [5].

Depending on workload, add-on orders can entail substantial manual effort from clinical laboratory staff [1, 2, 4, 5]. In our study, add-ons comprised 3.3 % of overall test volume, a figure that is very close to one previous study [4] and higher than another study [1]. Some laboratories, including ours, have implemented robotic specimen archival/retrieval units. As we have shown, the estimated impact of this type of unit on manual time can be substantial, with an estimated reduction of 24.1 hrs/day of manual add-on processing time in handling add-on order requests. However, the timing of add-on orders suggests that improvements on this design may include a combination of short-term/rapidly accessible and longer-term/less accessible specimen storage. Storage of specimens in a rapidly accessible buffer (e.g., very close to the chemistry analyzers) for a limited period of time (e.g., between 8 and 24 hours) would capture the majority of add-on orders. After this time period, specimens can be archived for longer-term storage in a space more distant from the instruments. At that point, turnaround time is likely less important.

The main limitations of our study are that the analysis is retrospective and confined to an academic medical center. The results may not generalize to other hospital or clinic settings. Nevertheless, it is hoped that the results described here provide useful to other institutions attempting to manage the challenges of add-on testing.

## Conclusions

Add-on orders significantly impact the workload of the clinical laboratory. In this study at an academic medical center, the majority of add-on orders were clinical chemistry tests, and most add-on orders occur within 24 hours of original specimen collection. Robotic specimen archival/retrieval units can reduce manual effort in the clinical laboratory associated with add-on orders.

## Abbreviations

A1C: Hemoglobin A1C
BILD: Direct bilirubin
BMP: Basic metabolic panel
CBC: Complete blood count
CPOE: Computerized provider order entry
CRP: C-reactive protein
DIFF: White blood count differential
ED: Emergency department
EHR: Electronic health record
ESR: Erythrocyte sedimentation rate
HAPT: Haptoglobin
HBSG: Hepatitis B surface antigen
HEPC: Hepatitis C antibody
ICU: Intensive care unit
LDH: Lactate dehydrogenase
LFP: Liver function panel
LIS: Laboratory information system
OSMO: Serum/plasma osmolality
PO4MG: Phosphorus and magnesium
PREALB: Prealbumin
PT/INR: Prothrombin time/international normalized ratio
PTT: Partial thromboplastin time
TAP: Toxic alcohol panel
UIHC: University of Iowa Hospitals and Clinics
URIC: Uric acid
